# Comparative Efficacy of Silver Diamine Fluoride and Sodium Fluoride in Managing Early Childhood Caries: An Updated Systematic Review and Meta‐Analysis

**DOI:** 10.1155/ijod/9959261

**Published:** 2026-02-24

**Authors:** Atiyeh Farahi, Nazieh Abdollah Kookhi, Benika Abbasi, Morteza Sharifi, Majedeh Nami, Yousef Moradi

**Affiliations:** ^1^ Department of Restorative Dentistry, School of Dentistry, Shahid Beheshti University of Medical Sciences, Tehran, Iran, sbmu.ac.ir; ^2^ Department of Prosthodontics, School of Dentistry, Kurdistan University of Medical Sciences, Sanandaj, Iran, muk.ac.ir; ^3^ Department of Prosthodontics, School of Dentistry, Isfahan University of Medical Sciences, Isfahan, Iran, mui.ac.ir; ^4^ Department of Prosthodontics, School of Dentistry, Hormozgan University of Medical Sciences, Bandarabbas, Iran, hums.ac.ir; ^5^ Health Metrics and Evaluation Research Center, Research Institute for Health Development, Kurdistan University of Medical Sciences, Sanandaj, Iran, muk.ac.ir

**Keywords:** early childhood caries, evidence synthesis, silver diamine fluoride, sodium fluoride

## Abstract

**Background:**

Early childhood caries (ECC) is a prevalent condition in preschool children that can impair nutrition and development. Comparative evidence on silver diamine fluoride (SDF) versus sodium fluoride (NaF) for arresting ECC remains limited.

**Objectives:**

To compare the efficacy of topical SDF and NaF in arresting ECC lesions and to evaluate effects on decayed, missing, and filled surfaces (DMFS).

**Data Sources:**

PubMed (Medline), Scopus, Embase, Web of Science, Cochrane Library, and ClinicalTrials.gov were searched from January 1990 through April 2024. Reference lists of included studies were hand‐searched.

**Eligibility Criteria:**

Randomized controlled trials (RCTs) directly comparing topical SDF and NaF in children aged 0–6 years with ECC and reporting outcomes for lesion arrest or DMFS.

**Study Selection and Data Extraction:**

Two reviewers independently screened records, extracted data, and assessed risk of bias using RoB 2. Discrepancies were resolved by a third reviewer.

**Number of Studies and Participants:**

Nine RCTs met inclusion criteria (total sample size reported in main text).

**Synthesis Methods:**

Random‐effects meta‐analysis (DerSimonian–Laird) was used to pool odds ratios (ORs) with 95% confidence intervals (CIs). Heterogeneity was assessed with *I* ^2^ and Cochran’s Q; publication bias was evaluated with Egger’s test. Preplanned subgroup analyses examined follow‐up duration.

**Results:**

Pooled analysis showed higher odds of caries arrest with SDF compared to NaF (OR 1.41; 95% CI 1.03 –1.94), while no statistically significant difference was observed for DMFS (OR 1.07; 95% CI 0.70–1.64). Heterogeneity across analyses was low to moderate, and risk‐of‐bias assessments identified some concerns in several trials. According to the GRADE evaluation, the certainty of evidence was rated moderate for caries arrest (downgraded for inconsistency) and low for DMFS (downgraded for risk of bias and imprecision).

**Conclusions:**

Compared with NaF, 38% SDF increases the likelihood of arresting ECC lesions while producing similar effects on DMFS. Clinical selection should balance lesion depth, aesthetic considerations, and patient‐level factors.

## 1. Introduction

Early childhood caries (ECC) is a major global public health concern, particularly affecting young children [1, 2]. When dental caries in primary teeth progresses to an advanced stage, it can lead to discomfort, infection, and long‐term problems with oral health. ECC is defined as the presence of decayed, missing, or filled primary tooth surfaces in children under the age of six, and it remains a prevalent condition worldwide [3, 4]. According to the Global Burden of Diseases report, untreated dental caries in primary teeth affected ~532 million children in 2017, making it the tenth most common disease globally. In regions like Egypt, the prevalence of ECC is alarmingly high, with 74% of children affected, underscoring the urgent need for effective preventive and management strategies [5].

Topical fluoride applications are central to caries prevention and management because they strengthen enamel and support remineralization [6–8]. Two topical agents commonly used in pediatric dentistry are silver diamine fluoride (SDF) and sodium fluoride (NaF). SDF combines antimicrobial silver with fluoride and has been promoted for its capacity to inhibit cariogenic bacteria and arrest lesion progression [9–13]. NaF, available in varnishes and gels, primarily enhances enamel resistance to demineralization [6, 8, 14, 15].

While some studies suggest that SDF may be more effective than NaF in halting carious lesions, others have found inconsistent results and highlighted specific downsides of using SDF, such as cosmetic concerns about tooth discoloration. For example, Mei and colleagues demonstrated that the efficacy of SDF in inhibiting demineralization and promoting the preservation of demineralized collagen in dentin is equivalent to 38%. These findings were demonstrated by forming a protective layer inside and within dentinal tubules under laboratory conditions [9]. Recent findings suggest that SDF could be an effective preventive protocol and potentially a suitable alternative to conventional NaF [16–18]. Additionally, SDF has been shown to successfully halt existing cavities. A comparative study published in the NCBI discovered that SDF administration for as little as 30 s was capable of dramatically arresting active caries lesions, with no statistically significant difference in efficacy when compared to lengthier 120 second applications [19]. However, this necessitates a thorough and comprehensive assessment of the clinical efficacy of each drug in controlling dental caries. Despite the widespread use of SDF and NaF in clinical practice, there is ongoing debate concerning their usefulness in treating ECC lesions. Several systematic reviews have been conducted by researchers such as Seifo et al. [20], Oliveira et al. [21], Dhanapriyanka et al. [22], Trieu et al. [23], and Rajendra et al. [24] to examine the comparative efficacy of SDF and NaF in the management and arrest of dental caries. These reviews have made significant contributions to our understanding of the roles of SDF and NaF in dental caries management. However, it is important to note that recent clinical trials and updated data, including studies up to April 2024, necessitate a more current and focused analysis. These newer studies provide data that could potentially impact the conclusions drawn regarding the relative efficacy of SDF versus NaF.

Previous reviews frequently pooled SDF with a variety of comparators or combined heterogeneous study designs, which limited the ability to draw direct conclusions about SDF versus NaF. This meta‐analysis restricts inclusion to trials that directly compare SDF and NaF, enabling a more focused assessment of relative efficacy. Both qualitative synthesis and quantitative meta‐analysis were performed, with primary emphasis on lesion arrest and decayed, missing, and filled surfaces (DMFS), outcomes that were inconsistently reported in earlier reviews. By applying strict inclusion criteria and standardized outcome measures, the analysis produces more nuanced and statistically robust estimates of comparative effect.

## 2. Materials and Methods

This study followed the Preferred Reporting Items for Systematic Reviews and Meta‐Analyses (PRISMA) guidelines and was preregistered with PROSPERO (CRD42024524613) [25].

### 2.1. Eligibility Criteria

Randomized controlled trials (RCTs) that directly compared topical SDF with NaF for the management of ECC were eligible. The target population comprised children with ECC aged 0–6 years. Primary outcomes of interest were caries arrest and DMFS. Excluded study types included animal research, in vitro studies, cross‐sectional designs, narrative reviews, editorials, conference abstracts, and trials that compared SDF or NaF with interventions other than placebo or standard care. Studies with incomplete outcome reporting or populations outside the specified age range were also excluded. Detailed inclusion and exclusion criteria are summarized in Table 1.

**Table 1:** PICOT structure, keywords, eligibility criteria, and search syntaxes.

Population	Childhood	
Intervention	Silver diamine fluoride (SDF), diamine silver fluoride, and silver ammonia fluoride	
Comparison	Sodium fluoride; sodium fluorides	
Primary outcome	Arresting caries; arresting dental caries; decayed, missing, and filled permanent teeth or surfaces (DMFS)	
Type of studies	Randomized control trials (RCTs)	
Inclusion criteria	Human studies published in English Randomized controlled trials Children with dental caries Studies comparing SDF with NaF Reporting at least one primary outcome (caries arrest and dental caries indices) Availability of full text	
Exclusion criteria	No full text available Outcomes or effect sizes not reported Nonhuman or laboratory studies Noncomparative studies (case reports, letters, and editorials)	
Mesh terms	Intervention (SDF): • *“Silver diamine fluoride”, “diamine silver fluoride”, “silver ammonia fluoride”* Comparison (NaF): • *“Sodium fluoride”, “sodium fluorides”* Condition (ECC): • *“Early childhood caries”, “dental caries”* Outcome: • *“Caries arrest”, “caries prevention”, DMFS*	
Search strategy sample	PubMed	(("silver diamine fluoride"[MeSH Terms] OR "silver diamine fluoride"[Title/Abstract] OR "diamine silver fluoride"[Title/Abstract] OR "silver ammonia fluoride"[Title/Abstract]) AND ("sodium fluoride"[MeSH Terms] OR "sodium fluoride"[Title/Abstract] OR "sodium fluorides"[Title/Abstract]) AND ("early childhood caries"[MeSH Terms] OR "early childhood caries"[Title/Abstract] OR "dental caries"[MeSH Terms] OR "dental caries"[Title/Abstract]) AND ("caries prevention"[MeSH Terms] OR "caries prevention"[Title/Abstract])) AND ("humans"[MeSH Terms])
	Scopus	(TITLE‐ABS‐KEY ("silver diamine fluoride") OR TITLE‐ABS‐KEY ("diamine silver fluoride") OR TITLE‐ABS‐KEY ("silver ammonia fluoride") AND TITLE‐ABS‐KEY ("sodium fluoride") OR TITLE‐ABS‐KEY ((fluoride AND sodium)) OR TITLE‐ABS‐KEY ((fluorides AND sodium)) OR TITLE‐ABS‐KEY ("Sodium Fluorides") AND TITLE‐ABS‐KEY ("early child caries") OR TITLE‐ABS‐KEY ("early childhood caries"))
	Web of Sciences	1# "sodium fluoride" (Topic) or (fluoride AND sodium) (Topic) or (fluorides AND sodium) (Topic) or "Sodium Fluorides" (Topic) 2# "early child caries" (Topic) or "early childhood caries" (Topic) 3# “silver diamine fluoride” (Topic) or “diamine silver fluoride” (Topic) or “silver ammonia fluoride” (Topic)
	Cochrane	(("silver diamine fluoride"):ti,ab,kw) AND (("sodium fluoride"):ti,ab,kw) AND (("early childhood caries" OR "dental caries"):ti,ab,kw) AND (("caries prevention"):ti,ab,kw) [Limit to: (Document Types Cochrane Reviews, Cochrane Trials) and (Publication Date from 1990 to 2023)]
	Embase	1‐ ‘silver diamine fluoride’/exp OR ‘silver diamine fluoride’:ab,ti 2‐ ‘sodium fluoride’/exp OR ‘sodium fluoride’:ab,ti 3‐ ‘early childhood caries’/exp OR ‘early childhood caries’:ab,ti OR ‘dental caries’/exp OR ‘dental caries’:ab,ti 4‐ ‘caries prevention’/exp OR ‘caries prevention’:ab,ti 5‐ 1 AND 2 AND 3 AND 4 6‐ [english]/lim 7‐ [article]/lim OR [review]/lim 8‐ [1990‐2023]/py 5 AND 6 AND 7 AND 8

### 2.2. Information Sources and Search Strategy

A comprehensive search strategy was developed using keywords and controlled vocabulary for “silver diamine fluoride,” “sodium fluoride,” and “early childhood caries,” together with relevant synonyms. Boolean operators (AND, OR) and database‐specific thesauri (MeSH, Emtree) were applied to optimize retrieval. Electronic searches covered PubMed (Medline), Scopus, Embase, Web of Science, the Cochrane Library, and ClinicalTrials.gov for records published from January 1990 through April 2024. Reference lists of included studies and relevant reviews were hand‐searched to identify additional reports. The full search strategy is provided in Table 1.

### 2.3. Study Selection

All retrieved records were imported into a reference management software, and duplicates were removed. Two reviewers (AF and NK) independently screened titles and abstracts against the eligibility criteria. Full‐text articles of potentially relevant studies were then retrieved and assessed independently by the same reviewers. Any disagreements were resolved through discussion, and if consensus could not be reached, a third reviewer (YM) adjudicated the decision. The study selection process is illustrated in a PRISMA flow diagram.

### 2.4. Data Extraction

A standardized data extraction form was developed prior to the review. The following data were extracted independently by two reviewers: author, year, study design, population characteristics, intervention and comparison details, outcomes, and key findings. Discrepancies were resolved by discussion, with consultation from a third reviewer if necessary. Data were entered into a predesigned spreadsheet to ensure accuracy and consistency.

### 2.5. Risk of Bias

Risk of bias for each included trial was assessed using the Cochrane Risk of Bias tool for randomized trials (RoB 2). The following domains were evaluated: randomization process, deviations from intended interventions, missing outcome data, measurement of the outcome, and selection of the reported result. Each domain received a judgment of low, some concerns, or high risk of bias, and an overall risk‐of‐bias judgment was assigned for each study [26].

### 2.6. Statistical Analysis

Effect sizes were expressed as odds ratios (ORs) with 95% confidence intervals (CIs) for dichotomous outcomes (caries arrest and DMFS events). Event counts were derived from reported group percentages at the end of follow‐up when raw counts were not directly provided. Pooled estimates were calculated using a random‐effects model (DerSimonian–Laird method) to account for between‐study variability. Heterogeneity was assessed using Cochran’s Q and quantified with the *I*2 statistic. Values of *I*2 were interpreted according to established thresholds: 0% – 25% (low), 25% – 50% (moderate), 50% – 75% (substantial), and  >75% (very high). Publication bias was assessed with Egger’s test. Preplanned subgroup analyses examined effects by follow‐up duration (months). Statistical significance was set at *p* < 0.05. Analyses were performed in STATA (version 17), and RevMan (version 5) was used to generate risk‐of‐bias summaries and forest plots. Certainty of evidence for each outcome was assessed using the Grades of Recommendation, Assessment, Development, and Evaluation (GRADE) approach, considering risk of bias, inconsistency, indirectness, imprecision, and publication bias. Two reviewers independently rated the certainty of evidence, with disagreements resolved by consensus. Evidence was classified as high, moderate, low, or very low according to GRADE Working Group criteria.

## 3. Results

### 3.1. Qualitative Results

**Figure 1:**
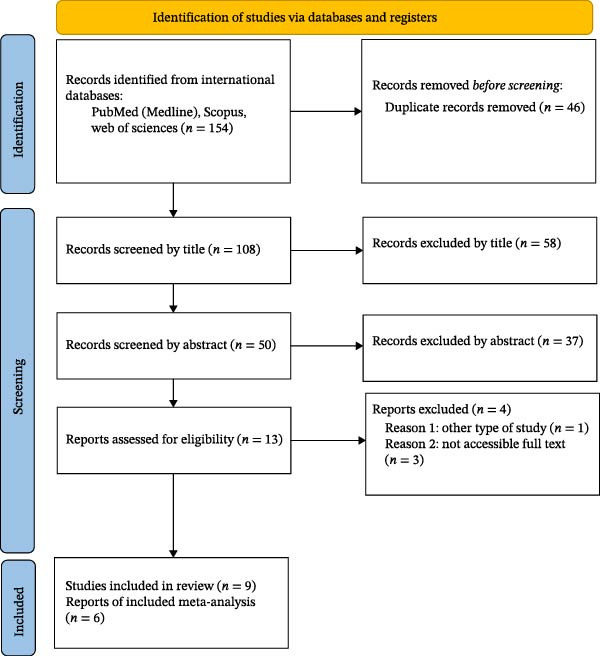
PRISMA 2020 flow diagram for new systematic reviews which included searches of databases and registers only.

In this review and analysis study, 9 clinical trials [27‐35] were included in the final meta‐analysis, after applying search and screening methods to titles, abstracts, and full text, with both arms receiving SDF and NaF, and focusing on various outcomes including DMFS and arrest (Figure 1 and Table 2). Overall, there were 2213 participants in the SDF group and 1053 in the NaF group, making up the total sample size. The SDF group consistently used doses equal to 38% of units in all selected studies, while the NaF group consistently used doses of 5% in all studies. The studies looked at children of different ages, ranging from 1 to 6 years old. The published studies spanned from 2017 to April 2023. Most studies typically had a follow‐up period lasting from 6 to 18 months, with the majority specifically having a 12‐month follow‐up (Table 2)[27‐35].

**Table 2:** The charactistics of included and selected studies.

Author/year (REF)	Design	Population	Duration	Sample	Randomization	Groups	Diagnostic criteria	Reporting result
Abdel Rehim et al. [34]	RCT	Children were recruited from outpatient clinic of Pediatric Dentistry and Dental Public Health Department, Faculty of Dentistry, Cairo University	All the children were recalled after 3, 6, 9, and 12 months	SDF = 31 NaF = 31	Simple randomization	Group I: (38%) Silver diamine fluoride applied every 6 months Group II: (5%) Sodium fluoride varnish applied every 3 months In group I, reapplication was performed every 6 months, while in group II it was performed every 3 months. Also, SDF was applied to any new lesion appeared during follow‐up periods	Assess progress of new caries, (tactile examination) and photographs, postoperative pain (detailed questionnaire), and related failure (abscess, pain, infection, and swelling) in both groups	12 months Pain SDF: 2/7% NaF: 8/6% New‐caries SDF: 0/6% NaF: 8/5% Failure SDF: 2/6% NaF: 8/5%
Abdellatif et al. [33]	RCT	Children aged ≤4 years from 4 nurseries in a rural area in Alexandria, Egypt joined the study in March 2022‐y had at least one active carious lesion with ICDAS codes ≥3	6‐months follow‐up	Control group: 110 Test group: 110		Control group: 38% SDF solution Test group: SDF + NaF	ICDAS	Lesion arrest rates Control group: 73/2 Test group: 77/7
Golpak/2020 [27]	RCT	Children aged 2–5 years with at least one carious lesion	12 months	SDF: 52 NaF: 52	Randomized by computer‐generated numbers by a secondary supervisor	Group 1: 38% SDF Group 2: 5% NaF varnish	DMFT	Mean DMFS (SD) SDF: 11.7 (8.4) NaF: 14.5 (12.4)
Mabangkhru et al./ 2020 [28]	RCT	Children aged 1−3 years who had at least one active dentin carious lesion	12 months	Group 1: 153 Group 2: 149	Stratified block randomization method	Group 1 = 38% SDF (Topamine), and Group 2 = 5% NaF varnish (Duraphat). Both agents were applied every 6 months onto the carious surface. 6‐ and 12‐month follow‐ups	Visible plaque index (VPI)/The decayed, missing, and filled teeth (DMFT) index/The carious lesion activity was evaluated by visual‐tactile inspection	Caries arrest rates in 12 months: Group 1: 35/7 Group 2: 20/9
Phonghanyudh et al./2022 [29]	RCT	children aged 1–3 years who had at least one active carious surface	18 months	Group 1: 147 Group 2: 143	Stratified block randomization	Group 1: 38% SDF Group 2: 5% NaF varnish 6, 12, and 18‐month follow‐up examinations	ICDAS Parental satisfaction with the dental appearance of children at baseline and 18‐month follow‐up and between the intervention groups were compared using the McNemar test and chi‐square test, respectively	Caries arrest rate in 18 months: Group 1: 59/1 Group 2: 58/8
Sirivichayakul et al./2023 [35]	RCT	Preschool children were recruited if they had at least one initial approximal carious lesion at the distal surface of the canines, both approximal surfaces of the first molars, or the mesial surface of the second molars assessed from bitewing radiographs	18 months	Group 1 (control group): 64 Group 2 (semi‐annual NaF varnish application): 62 Group 3 (semi‐annual SDF application): 64	Stratified block randomization	Group 1 (placebo control), Group 2 (5% sodium fluoride [NaF] varnish), and Group 3 (38% silver diamine fluoride [SDF]). All agents were applied semiannually	dmft/dmfs The plaque score was measured on a scale of 0–3	Caries development rate in 18 months: Group 1: 218 (24.1) Group 2: 149 (17.1) Group 3: 247 (27.2)
Yassin et al./2023 [31]	RCT	Children aged ≤ 4 years old with at least one active carious lesion (ICDAS score ≥ 3)	3 months	Group 1:82 Group 2:83	Participants were randomly assigned in a 1:1 ratio using a computer‐generated list of random numbers	Group 1: 38% SDF Group 2: 5% NaF varnish combined with 2 Motivational Interviewing sessions, at baseline and after three months	ICDAS‐LAA criteria	Caries arrest: Group 1: 63.7% Group 2: 58.1%
Thwin et al./2017 [30]	RCT	Five preschools were selected for the study based on the census sampling method by the Department of Social Welfare, Ministry of Social Welfare, Relief and Resettlement	6 months	Group 1: 177 Group 2: 79		Group 1: SDF and sodium fluoride (NaF) Group 2: only NaF application	DMFT, DMFS, the caries risk test was performed by using Dentocult® SM (Oral Care Co. Ltd., Japan) and Cariostat® (DENTSPLY‐Sankin K.K. Co., Ltd., Japan)	Group 1: DMFT: 5.76 ± 4.67 DMFS: 11.63 ± 13.93 Group 2: DMFT: 6.07 ± 4.76 DMFS: 12.42 ± 12.12
Zheng et al./2023 [32]	RCT	y 3‐ to 4‐year‐old children	12 Months	Group 1: 344 Group 2: 344	Stratified block randomization	Group 1: SDF 38% Group 2: Sodium fluoride varnish	DMFT and DMFS	Mean new decayed surface: Group 1: 0.4 § 1.5 (*n* = 209) Group 2: 0.4 § 1.3 (*n* = 225)

### 3.2. Quantitate Results

Quantitative analysis was conducted on certain studies to evaluate specific results such as caries, failure, filling, or overall DMFS index, as well as the outcome of arrest rate in the SDF versus NaF group. After retrieving the pertinent information, the findings indicated that only the Abdel Rehim et al. [34] study evaluated the mean pain or percentage reduction in pain. In the other selected studies, the mean pain or percentage of pain reduction in both groups was not individually reported. Hence, this index was not examined because of the limited quantity of studies.

#### 3.2.1. First Outcome: Odds of DMFS

In relation to the measures of caries and failure research also presented varying findings. For instance, regarding failure, only the study by Abdel Rehim et al. [34] observed this result in two groups after 12 months of follow‐up. Furthermore, the study found cavities in both groups that were given SDF and NaF over a 12‐month period. Golpak [27] and colleagues observed decay in both groups after 6 and 12 months, but no instances of failure were documented in the research. Research conducted by A. Phonghanyudh et al. also explored the decay percentage over 9, 12, and 18 months of follow‐up. Research conducted by Mabangkhru et al. [28] and colleagues examined the expected results in each group based on the percentage of DMFS/T cases after 6 and 12 months.

**Figure 2:**
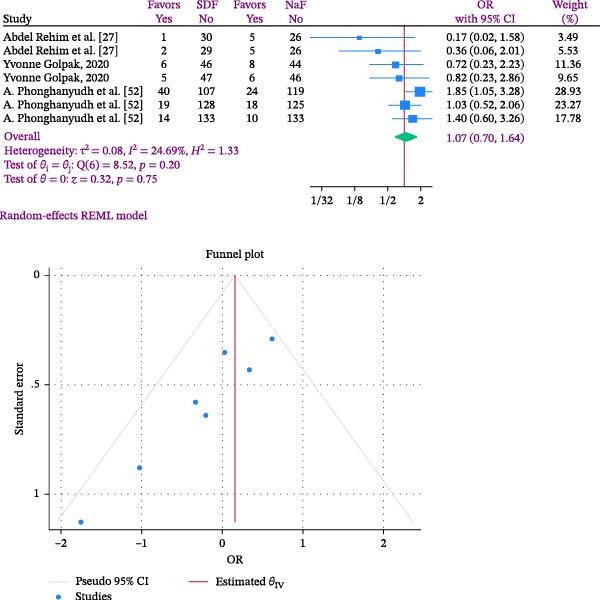
Forest and Funnel plots comparing SDF and Naf in the odds of developing DMFS in children.

Taking into account the varying results from the chosen studies, the authors opted to acknowledge and present all of these results in the format of DMFS. According to this explanation, the odds of developing DMFS in those who received SDF was 1.07 times higher than in those who received NaF, as shown in the results of the meta‐analysis (OR: 1.07; 95% CI: 0.70–1.64; I2:24.69%; *p* heterogeneity: 0.20). There was a 0.07% higher likelihood of DMFS happening in the SDF group compared to NaF recipients; however, this result did not show statistical significance (Figure 2). In Figure 2, the study by Phonghanyudh et al. [29] showed the highest effect size after an 18‐month follow‐up, while the study by Abdel Rehim et al. [34] had the lowest effect size after a 12‐month follow‐up.

The analysis showed a heterogeneity rate of 24.69% with a *p* Value of 0.20. The heterogeneity rate was minimal and indicated consistency among the studies. Publication bias was assessed using Egger’s regression test. Due to the limited number of included trials, a funnel plot could not be reliably generated. The Egger’s test results (B = −2.57; SE = 0.957; *p* = 0.0072) indicated potential publication bias, raising concern that smaller studies with nonsignificant or unfavorable findings may be underrepresented (Figure 2).

**Figure 3:**
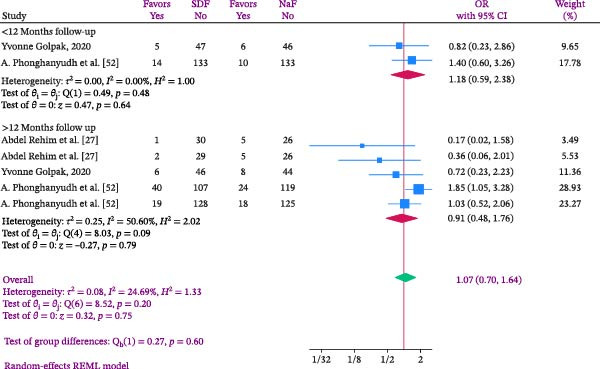
Forest plot comparing SDF and Naf in the odds of developing DMFS in children according to follow‐up time.

Analysis within subgroups was conducted according to the length of the follow‐up period, with the findings presented in Figure 3. Subgroup analysis was categorized into less than 12 months and more than 12 months based on follow‐up duration. The results indicated that the likelihood of DMFS occurrence in SDF recipients was 1.18 times greater than those receiving NaF (OR: 1.18; %95 CI: 0.59–2.38; I2:0.00%; *p* heterogeneity: 0.48). For follow‐up periods exceeding 12 months, this effect decreased to 0.91 (OR: 0.91; %95 CI: 0.48–1.76; I2:50.60%; *p* heterogeneity: 0.09), although neither were statistically significant (Figure 3). The studies typically provided the age range of the children based on different age groups, without specifying the exact age at which the children were observed. Hence, no subgroup analysis was conducted according to age.

#### 3.2.2. Second Outcome: Odds of Arrest Rate

**Figure 4:**
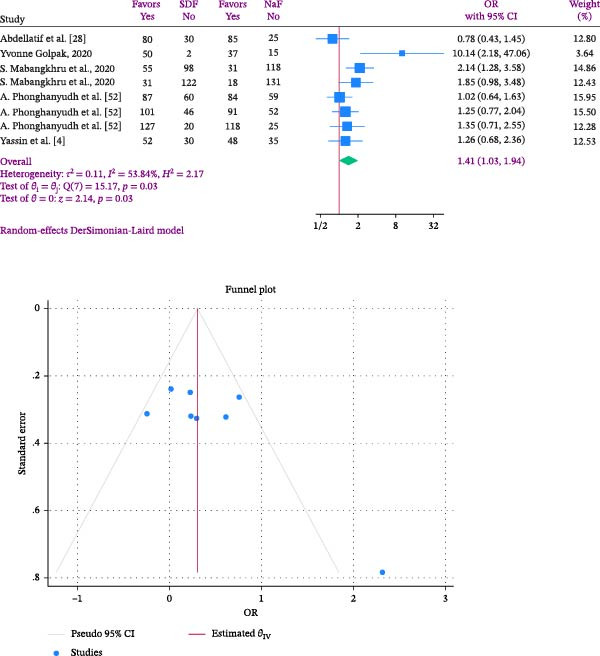
Forest and Funnel plots comparing SDF and Naf in the odds of arrest rate in children.

A meta‐analysis indicated that individuals in the SDF group had a 1.41 times higher chance of experiencing arrest rate compared to those in the NaF group (OR: 1.41; %95 CI: 1.03–1.94; I2:53.84%; *p* heterogeneity: 0.03). This indicates that the chance of being arrested in the SDF group was 41% more than NaF users, with statistical significance (Figure 4). The Golpak [27] study showed the highest effect size after 12 months follow‐up, while the Abdellatif et al. [33] study had the lowest effect size after 6 months follow‐up (Figure 4).

In this study, heterogeneity was quantified at *I* ^2^ = 53.84% with a corresponding *p* value of 0.03. According to conventional thresholds, this represents substantial heterogeneity, indicating that more than half of the variability across trials reflects genuine differences rather than random error. While the overall direction of effect was consistent, this level of heterogeneity suggests that pooled estimates should be interpreted with caution, as clinical and methodological diversity may have influenced outcomes. To assess publication bias, Egger’s regression test was performed. Due to the limited number of included studies, a funnel plot could not be reliably generated. The Egger’s test results (B = 3.50; SE = 1.523; *p*  = 0.0216) suggested potential publication bias, raising concern that smaller studies with null or unfavorable findings may be underrepresented. This bias could exaggerate the apparent benefit of SDF, thereby reducing confidence in the pooled estimates.

**Figure 5:**
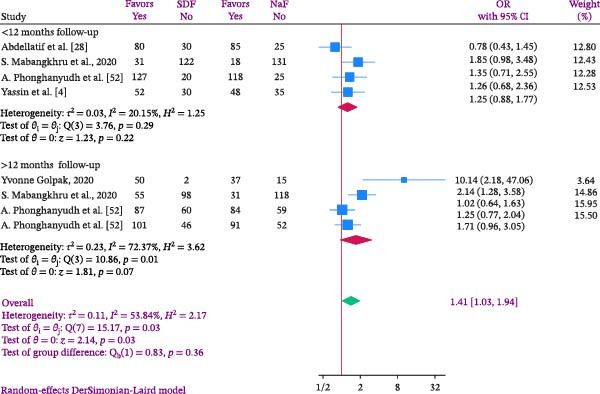
Forest plot comparing SDF and Naf in the odds of arrest rate in children according to follow up time.

Based on the length of the follow‐up period, subgroup analysis was conducted, and the findings were presented in Figure 5. Subgroup analysis categorized participants into those followed for less than 12 months and those followed for over 12 months. The findings revealed that the risk of arrest rate in the SDF group was 1.25 times higher than in the NaF group for follow‐up periods under 12 months and 1.71 times higher for follow‐up periods exceeding 12 months. However, none of these differences were statistically significant (Figure 5). The studies usually indicated the age range of the children based on their age groups, but did not specify the exact ages of the children being examined. Hence, age‐based subgroup analysis was not conducted.

#### 3.2.3. Sensitivity Analysis

##### 3.2.3.1. DMSF

Leave‐one‐out influence analysis showed that omission of any single study did not materially change the pooled estimate (range OR 0.98–1.16), indicating no single study unduly drove the result. Results were robust to model choice (REML random‐effects OR 1.07, 95% CI 0.70–1.64) and to exclusion of trials judged at high risk of bias or of very small weight; *I* ^2^ and *τ* ^2^ remained low to moderate (Supporting information file, Figure S1A).

##### 3.2.3.2. Caries Arrest

Leave‐one‐out analysis showed that no single study unduly influenced the pooled estimate (range OR 1.28–1.46). The overall effect favoring SDF remained consistent across random‐effects (DerSimonian–Laird) and fixed‐effect models. Excluding studies with high risk of bias or low weight did not materially alter the result (Supporting file, Figure S1B).

### 3.3. Risk of Bias

**Figure 6:**
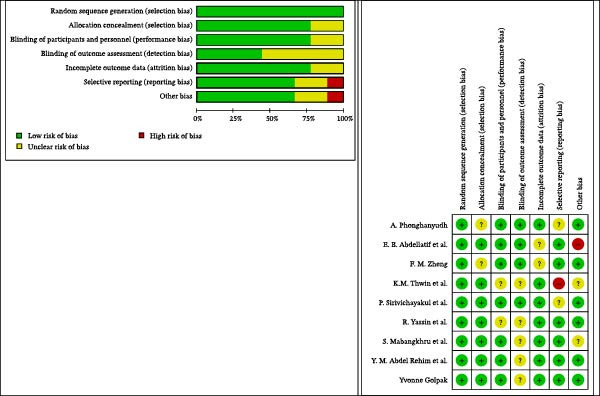
Risk of bias graph: review authors’ judgments about each risk of bias item presented as percentages across all included studies. Risk of bias summary: review authors’ judgments about each risk of bias item for each included study.

The quality assessment results revealed that a significant portion of the chosen studies in this meta‐analysis exhibit high quality and are categorized as having low levels of bias. The scales of bias have the fewest errors overall, but in the blinding of outcome assessment scale, nearly half of the intervention studies assessed have this bias labeled as unclear (Figure 6).

### 3.4. GRADE Results

The GRADE assessment indicated that the certainty of evidence for the DMFS outcome was low. This rating reflected concerns about risk of bias in several included trials and imprecision due to wide CIs, which limited confidence in the pooled estimate. In contrast, the certainty of evidence for caries arrest was rated as moderate. Although the pooled analysis demonstrated a statistically significant benefit of SDF compared to NaF, the presence of moderate heterogeneity across studies led to downgrading for inconsistency (Table 3).

**Table 3:** Summary of findings: silver diamine fluoride (38%) versus sodium fluoride (5%) for early childhood caries.

Patient or population: Children aged 1–6 years with early childhood caries Setting: Randomized controlled trials (2017–2023) Intervention: 38% Silver dDiamine fluoride (SDF) Comparison: 5% Sodium fluoride (NaF)				
Outcomes	Relative effect (95% CI)	№ of participants (studies)	Certainty of the evidence (GRADE)	Comments
DMFS	OR 1.07 (0.70–1.64)	2,213 SDF vs. 1,053 NaF (9 RCTs)	⨁⨁◯◯ Low	No significant difference; downgraded for risk of bias and imprecision
Caries arrest	OR 1.41 (1.03–1.94)	2,213 SDF vs. 1,053 NaF (9 RCTs)	⨁⨁⨁◯ Moderate	Favors SDF; downgraded for inconsistency (heterogeneity)

*Note:* GRADE Working Group certainty ratings: High: very confident in the estimate. Moderate: moderately confident, true effect likely close but may differ. Low: limited confidence, true effect may differ substantially. Very low: very little confidence, true effect likely substantially different.

Abbreviations: CI: confidence interval; OR: odds ratio.

^∗^The risk in the intervention group (and its 95% confidence interval) is based on the assumed risk in the comparison group and the relative effect of the intervention (and its 95% CI).

## 4. Discussion

Tooth decay is a multifactorial, noncommunicable condition that can compromise nutrition and growth in young children [27]. Beyond the discomfort and illness caused by ECC, poor oral health can have a significant impact on children’s nutritional intake, hence influencing their growth and development [29–31]. Management of ECC is particularly challenging in very young or uncooperative patients, where conventional restorative care may require sedation or general anesthesia. As a result, researchers should create evidence‐based methodologies to look for less intrusive effective management techniques, such as SDF varnish and NaF application, as a conservative strategy to treating caries without requiring general anesthesia [31, 32]. Pooled estimates indicated no statistically significant difference in DMFS between SDF and NaF, while SDF demonstrated greater odds of lesion arrest (OR 1.41). SDF increases biofilm pH, reduces dentin demineralization, and exerts antibacterial effects; the 38% formulation predominates in clinical practice and the literature. It is frequently available in a 38% solution with around 255,000 ppm silver and 44,800 ppm fluoride ions. Review publications have indicated a 38% concentration intervention for the prevention and treatment of dental caries in children [31, 33–36]. The moderate heterogeneity observed (*I* ^2^ = 53.84% ) implies that variability among included trials was not solely due to chance. Differences in follow‐up duration, outcome definitions, and study populations may have contributed to this inconsistency. Clinically, this means that while the overall direction of effect favors SDF for caries arrest, the magnitude of benefit may vary across settings, underscoring the need for context‐specific interpretation. The analysis restricted inclusion to studies using 38% SDF to improve internal consistency, while acknowledging that other concentrations may yield different effects. The concentration of 38% is widely employed in both clinical practice and research, demonstrating effectiveness in the arrest and management of caries. However, it is important to acknowledge that SDF is available in alternative concentrations, such as 12% and 30%, which may yield different outcomes. The exclusion of studies employing other concentrations potentially restricts the generalizability of the findings. Future investigations would benefit from the inclusion of studies utilizing diverse SDF concentrations, thereby offering a more comprehensive understanding of its efficacy across various formulations. SDF demonstrated greater odds of lesion arrest (OR 1.41) compared with NaF in the pooled randomized trials. This indicates a substantial benefit of SDF in halting the progression of ECC. Conversely, the slight increase in DMFS observed with SDF compared to NaF suggests that while SDF is effective in arresting caries, its impact on overall caries experience (as measured by DMFS) is less pronounced. The variability in effect sizes among studies highlights the importance of considering study design, sample characteristics, and treatment protocols in interpreting the results. While SDF showed a statistically significant advantage over NaF in arresting caries, the GRADE assessment rated this evidence as moderate certainty, downgraded for inconsistency due to heterogeneity across trials. For DMFS, the certainty of evidence was low, reflecting unclear allocation concealment in several studies and wide confidence intervals. These ratings indicate that although the direction of effect is consistent, confidence in the magnitude of benefit is limited.

Mechanistic and laboratory studies support SDF’s anticaries properties: increased biofilm pH, reduced dentin demineralization, and antibacterial activity against cariogenic species [37]. Previous comprehensive evaluations have found that silver compounds can help prevent and treat caries in both the primary and permanent dentitions [38–41]. Clinically, SDF is recommended for high‐risk or difficult‐to‐treat lesions, for patients with behavioral or medical challenges, and in settings with limited access to restorative care because of its low cost, ease of application, and potential to reduce treatment‐related anxiety [41, 42].

Adverse effects associated with SDF include transient soft‐tissue irritation and tooth discoloration; pulpal toxicity has not been substantiated in clinical reports [40]. SDF has been reported to be harmless to the tooth pulp [41, 43]. One of the downsides of SDF treatment, according to some research, is the appearance of black teeth stains. An in vitro investigation found that adding potassium iodide to SDF during application reduced tooth discoloration [44]. This study implies that lower SDF concentrations may delay the beginning of adverse effects; nevertheless, these levels are less efficient at stopping caries. Ex vivo and in vivo investigations on cavitated removed teeth from children who received semiannual treatments of SDF revealed that it was efficient in halting lesions and increased fluoride uptake when compared to fluoride varnish and acidulated phosphate fluoride gel [45, 46]. It is recommended that SDF be used semiannually at a concentration of 38% [47].

Sodium fluoride varnish (commonly 5% NaF; ~22,600 ppm fluoride) is an effective professional topical agent for enamel remineralization and caries prevention. Fluoride varnish typically comprises 5% NaF as the active ingredient (22,600 ppm fluoride). NaF varnish inhibits caries, stops enamel lesions, and softens dentine caries [48]. A recent meta‐analysis found that using 5% NaF varnish resulted in 63.6% of remineralized enamel caries [49].

The meta‐analysis discovered that SDF had a slightly greater chance of DMFS than NaF, but this distinction did not show statistical significance. The research indicated that the SDF group had a much greater likelihood of caries arrest in comparison to the NaF group. Fluoride ions primarily influence mineral dynamics at the enamel surface, while silver ions exert antimicrobial effects that may be more relevant to dentin‐involved lesions; consequently, SDF’s advantage for arresting active lesions may not translate into a large difference in composite DMFS measures. According to Liu et al., a significant amount of fluoride was successful in preventing enamel demineralization, while using silver ions on their own had minimal impact [28, 50, 51]. It is possible that fluoride ions primarily impact the tooth substrate by causing demineralization and remineralization, while silver ions may mainly target cariogenic bacteria in dentine caries, rather than affecting the precipitation of mineral ions in enamel. The treatment had a greater effect on noncavitated enamel caries (ICDAS 2) compared to cavitated enamel caries (ICDAS 3). This emphasizes the significance of early intervention in effectively managing enamel caries. Tooth decay on the front teeth is usually easier to manage compared to decay on the back teeth. Caries on the outer or inner surface of the tooth enamel were more likely to be halted than caries on different surfaces of the tooth. These regions may be readily reached for cleaning and are in contact with saliva and fluoride, potentially enhancing the remineralization process. Another important discovery was that enamel decay in children who use fluoride toothpaste while brushing their teeth was more inclined to be stopped as opposed to those who use non‐fluoride toothpaste. This outcome underlines the significance of promoting parents and caregivers to brush their children’s teeth with fluoride toothpaste along with professionally applied topical fluorides.

Both 5% NaF varnish and 38% SDF showed comparable results in treating enamel caries, making them both suitable for halting its progression. 5% NaF varnish is a better option for children with only enamel caries to prevent black staining caused by 38% SDF. SDF with a concentration of 38% could be a better choice for children with both enamel and dentine caries and without aesthetic concerns compared to using 5% NaF varnish, as it has shown more effectiveness in stopping dentine caries [42, 52]. The finding that SDF significantly increased caries arrest but did not improve DMFS highlights an important distinction between lesion‐level and surface‐level outcomes. Arresting existing lesions does not necessarily reduce the cumulative DMFS score, particularly if new lesions develop or if arrested lesions remain classified as decayed. Moreover, although all studies used 38% SDF and 5% NaF, application frequency and duration varied (e.g., every 3 vs. every 6 months), introducing heterogeneity that may influence efficacy and comparability. These factors likely contributed to the moderate heterogeneity observed and underscore the need for standardized protocols and outcome definitions in future trials [23, 53].

This review was constrained by the relatively small number of eligible trials, which required the use of a composite DMFS outcome rather than analysis of individual components. Important contextual variables such as toothbrushing habits and baseline oral hygiene were not reported, limiting subgroup analyses. At the study level, most trials had short follow‐up periods, inconsistent outcome definitions, and limited reporting of patient‐centered outcomes. At the review level, restrictions to selected databases and English‐language publications may have excluded relevant studies. Egger’s test suggested possible publication bias, though the small number of studies reduces confidence in this finding. Overall, these limitations call for cautious interpretation and highlight the need for larger, well‐designed trials with standardized outcome reporting. To strengthen the evidence base, larger randomized trials with standardized outcome reporting, stratification by lesion severity and tooth surface, and evaluation of different SDF concentrations and application intervals are recommended. The GRADE evaluation highlights important differences in the strength of evidence across outcomes. Moderate‐certainty evidence supports the conclusion that SDF is more effective than NaF in arresting caries, although variability between studies suggests that the magnitude of effect may differ depending on follow‐up duration and study design. Overall, the GRADE findings emphasize that while SDF shows promise in arresting caries, the strength of evidence is not uniformly high, and additional robust trials are required to increase confidence in these results.

## 5. Conclusion

In conclusion, this meta‐analysis provides evidence supporting the superior efficacy of SDF over NaF in the arrestment of ECC. Specifically, SDF demonstrates a higher likelihood of caries arrest, while exhibiting a comparable effect on DMFS outcomes. These findings emphasize the significance of incorporating SDF as a treatment modality for the management of ECC. Nevertheless, the study also emphasizes the necessity for further research to investigate the impacts of varying SDF concentrations and to address potential biases and methodological limitations. Clinicians should carefully consider these factors when making decisions regarding caries management strategies and should continue to prioritize evidence‐based approaches to optimize patient outcomes.

## Author Contributions

**Yousef Moradi:** conceptualization, methodology, formal analysis, data curation, investigation, writing – original draft, writing – review & editing, supervision, project administration. **Atiyeh Farahi:** methodology, investigation, data curation, formal analysis, writing – original draft, visualization, project administration. **Nazieh Abdollah Kookhi:** investigation, data curation, writing – original draft, writing – review & editing, visualization. **Benika Abbasi:** investigation, data curation, formal analysis, writing – original draft, visualization. **Morteza Sharifi:** investigation, data curation, formal analysis, writing – original draft, writing – review & editing, and visualization. **Majedeh Nami:** investigation, data curation, writing – original draft, writing – review & editing, and visualization.

## Funding

No funding was received for this manuscript.

## Ethics Statement

The authors have nothing to report.

## Conflicts of Interest

The authors declare no conflicts of interest.

## Supporting Information

Additional supporting information can be found online in the Supporting Information section.

## Supporting information


**Supporting Information** Supporting Information provide additional robustness checks and sensitivity analyses for the main outcomes. Figure S1A (DMSF): Leave‐one‐out influence analysis and model comparisons confirmed stability of pooled estimates, with low to moderate heterogeneity. Figure S1B (Caries arrest): Results consistently favored SDF, with pooled estimates robust across analytic models and exclusion of high‐risk or low‐weight studies. Together, these supporting figures demonstrate that findings were not unduly driven by any single study or analytic choice, supporting the reliability of the main conclusions.

## Data Availability

The authors have nothing to report.
