# Supplementary material for: Comparative Efficacy of Silver Diamine Fluoride and Sodium Fluoride in Managing Early Childhood Caries: An Updated Systematic Review and Meta‐Analysis
Source: Int J Dent. 2026 Feb 24;2026:9959261. doi: 10.1155/ijod/9959261 (PMC12930214; doi:10.1155/ijod/9959261)
Supplement: Supplementary file 1 — Supporting Information Supporting Information provide additional robustness checks and sensitivity analyses for the main outcomes. Figure S1A (DMSF): Leave‐one‐out influence analysis and model comparisons confirmed stability of pooled estimates, with low to moderate heterogeneity. Figure S1B (Caries arrest): Results consistently favored SDF, with pooled estimates robust across analytic models and exclusion of high‐risk or low‐weight studies. Together, these supporting figures demonstrate that findings were not unduly driven by any single study or analytic choice, supporting the reliability of the main conclusions. [file IJOD-2026-9959261-s001.docx]

Sensitivity Analysis

A: DMFS

B: Arrest rate
